# Cancer associated-fibroblast-derived exosomes in cancer progression

**DOI:** 10.1186/s12943-021-01463-y

**Published:** 2021-12-01

**Authors:** Chao Li, Adilson Fonseca Teixeira, Hong-Jian Zhu, Peter ten Dijke

**Affiliations:** 1 Oncode Institute and Department of Cell and Chemical Biology, Leiden University Medical Center, Leiden, Netherlands; 2 Department of Surgery, The Royal Melbourne Hospital, The University of Melbourne, Parkville, VIC Australia

**Keywords:** TME, CAFs, cancer cells, immune cells, exosomes, biomarkers

## Abstract

To identify novel cancer therapies, the tumor microenvironment (TME) has received a lot of attention in recent years in particular with the advent of clinical successes achieved by targeting immune checkpoint inhibitors (ICIs). The TME consists of multiple cell types that are embedded in the extracellular matrix (ECM), including immune cells, endothelial cells and cancer associated fibroblasts (CAFs), which communicate with cancer cells and each other during tumor progression. CAFs are a dominant and heterogeneous cell type within the TME with a pivotal role in controlling cancer cell invasion and metastasis, immune evasion, angiogenesis and chemotherapy resistance. CAFs mediate their effects in part by remodeling the ECM and by secreting soluble factors and extracellular vesicles. Exosomes are a subtype of extracellular vesicles (EVs), which contain various biomolecules such as nucleic acids, lipids, and proteins. The biomolecules in exosomes can be transmitted from one to another cell, and thereby affect the behavior of the receiving cell. As exosomes are also present in circulation, their contents can also be explored as biomarkers for the diagnosis and prognosis of cancer patients. In this review, we concentrate on the role of CAFs-derived exosomes in the communication between CAFs and cancer cells and other cells of the TME. First, we introduce the multiple roles of CAFs in tumorigenesis. Thereafter, we discuss the ways CAFs communicate with cancer cells and interplay with other cells of the TME, and focus in particular on the role of exosomes. Then, we elaborate on the mechanisms by which CAFs-derived exosomes contribute to cancer progression, as well as and the clinical impact of exosomes. We conclude by discussing aspects of exosomes that deserve further investigation, including emerging insights into making treatment with immune checkpoint inhibitor blockade more efficient.

## Introduction

Cancer is a genetic disease in which multiple mutations in genomic DNA drive uncontrolled proliferation and cell morphological changes. Although cancer can be divided into different types according to its location and cell types, most cancers share a series of common characteristics: self-sufficiency in growth signals, limitless replicative potential, increased metastasis and invasion, insensitivity to antigrowth signals, resistance to cell death, activating angiogenesis, metabolic reprogramming and escaping immune surveillance [1]. Importantly, the mutations that functionally inactivate tumor suppressor gene products or activate proto-oncogene products are key factors that drive tumorigenesis [2].

Cancers can be described as a never healing wounds due to the integration of cellular activities and the role of inflammation and cytokines [3, 4]. The signaling pathways that initially are activated to repair the lesion are similar in both processes, i.e. cancer development and wound healing. The vital difference between cancers and wound healing is the sustainability and exacerbation of the signaling pathways in cancer cells and their microenvironment [5]. Cancer development is a very dynamic and multistep process, and cells within a cancer are heterogeneous of which the (relative) composition changes during disease progression. Not only, is there communication between cancer cells within a tumor, but also between multiple cell types in the tumor microenvironment (TME) [6, 7]. The TME is composed of various cell types, including stromal cells (e.g. fibroblasts, mesenchymal stromal cells, pericytes, and adipocytes) and immune cells (e.g. T and B lymphocytes, natural killer (NK) cells and tumor-associated macrophages (TAMs)); all these cells are embedded in the extracellular matrix (ECM) [8, 9]. While initially studies were focused on interfering with the malignant behavior of cancer cells for therapeutic intervention in cancer treatment, recently more research is geared towards the targeting of TME to find novel cancer therapies. In particular, immune therapy targeting immune checkpoint inhibitors (ICIs) has shown dramatic long-lasting beneficial effects, even on patients with metastatic disease [10]. However, immune therapy only works for about 15% of cancer patients [11]. Recently, a combination of ICIs with anti-angiogenic therapy has been developed to overcome the limitations of ICIs monotherapy, mainly by inhibiting angiogenesis and increasing the infiltration of cytotoxic T cells into the TME [12, 13]. This combination strategy targeting on TME has demonstrated more clinical benefits and promising outcomes in many clinical trials [14].

Among the multiple stromal cell types in the TME, the cancer associated fibroblasts (CAFs) are a dominant component [15] of several cancer types including breast, colon, pancreatic and prostate cancers [16, 17]. In pancreatic cancer, 60–70% of tumor mass is composed of stromal tissue characterized by CAFs and excessive collagen and other ECM component deposition [16, 18]. CAFs are a highly heterogeneous cell type, some subtypes of which have cancer-restraining and others have cancer-promoting properties [19]. Also, CAFs can be divided into several subtypes according to their differential expression of specific biomolecular markers, and different subtypes exert different functions [20]. Fibroblasts are usually quiescent in normal tissues, but they can be activated during tissue damage [19]. These activated fibroblasts located in the vicinity of cancer cells are an important subtype of CAF population [21]. CAFs modulate cancer development through multiple aspects such as influencing cancer cell invasion and metastasis, promoting immune evasion, stimulating angiogenesis and promoting chemotherapy resistance [22–25]. In addition to ECM remodeling, CAFs can exert great impact on tumorigenesis through paracrine factors, such as cytokines and exosomes (see below).

The intercellular communication occurs via multiple ways, such as by direct cell-cell contact and by the transfer of secreted molecules or vesicles. Secretion of exosomes is an important way for CAFs to influence behavior of cancer cells (and *vice versa*). Exosomes are a subtype of extracellular vesicles (EVs), which originate from endosomal vesicles secreted by cells [26]. Most exosomes are small (s) EVs and have a diameter between 30 nm to 150 nm [27]. These sEVs contain proteins, nucleic acids and lipids that can be transferred from one cell to another, and thereby (in) activate signaling pathways [28]. Exosomes secreted from cancer cells, immune cells and other cell types in the TME also exert great impact on tumorigenesis. These aspects have been excellently summarized by others and will not be the focus of our review [29–33]. Here, we concentrate on the role of CAFs-derived exosomes in cancer progression. Our review is structured as follows: we first introduce the role of CAFs in cancer development and how CAFs communicate with others cells in TME. Then, we describe on how CAFs-derived exosomes regulate cancer cells and other cells of TME. At last, as exosomes inform the heterogeneous biological processes related to tumor growth and have a therapeutic potential [28], we discuss the clinical applications of exosomes, in particular within the context of making immune checkpoint therapy more effective.

## The role of CAFs in cancer progression within the TME

### What are CAFs?

Fibroblasts act a critical role in connective tissues, maintaining tissue homeostasis by producing connective tissue ECM and different cytokines [34]. In healthy human tissues, they are usually quiescent as shown by their low levels of cell proliferation and metabolic activity [35]. Fibroblasts can be activated during tissue injury or inflammatory response, with enhanced cell proliferation and metabolic activity, including protein synthesis [36]. The activated fibroblasts observed in biological processes, such as wound healing and fibrosis, are called myofibroblasts, which express more fibroblast activation protein α (FAP) and α-smooth muscle actin (α-SMA) and incorporate α-SMA into cytoplasmic stress fibers [37, 38]. Compared with quiescent fibroblasts, myofibroblasts acquire contractile properties and secretory profiles that promote tissue repair during wound healing and cancer development [19].

CAFs are fibroblasts that are observed within the tumor microenvironment near cancer cells [15]. Generally, CAFs can be characterized through the expression of various mesenchymal markers, morphological features such as a spindle shape, and lack of expression of non-mesenchymal cell markers, such as markers for epithelial, endothelial, immune and neuronal cells. Activated CAFs are expected to express various marker proteins [25, 39], including α-SMA, FAP, fibroblast-specific protein 1 (FSP1), podoplanin (PDPN) and platelet-derived growth factor receptor (PDGFR) [24, 40]. However, these markers are not unique to CAFs; they are also expressed in other cell types and healthy tissues. Currently, there is no single marker to identify all CAF-subtypes or to differentiate CAFs from other cell types [19].

In addition to distinguishing CAFs from other cell types, CAFs may also be subcategorized in different populations. Based on their resemblance with activated fibroblasts that are observed in non-malignant lesions, CAFs with high expression of α-SMA are called myofibroblastic CAFs (myCAFs) [41]. However, CAFs are more heterogeneous than other fibroblasts and not all CAFs demonstrate elevated expression of α-SMA. Combined to histologic techniques and fluorescence activated cell sorting (FACS) analysis, the emerging use of single-cell RNA sequencing (scRNA-seq) have added important information regarding CAFs heterogeneity. Whereas no specific biomarker was established so far, similar phenotypes have been described in different types of cancer, reinforcing their relevance as important players during cancer progression. In addition to myCAFs, the inflammatory CAF (iCAF) is another phenotype described in pancreatic cancer, which demonstrates low α-SMA expression as opposed to classic “activated CAFs”. iCAFs can release high levels of inflammatory cytokines such as interleukin-6 (IL-6), interleukin-11 (IL-11) and leukemia inhibitory factor (LIF) and lead to the immune suppression [41, 42]. Similarly, the presence of CAFs with immunomodulatory function has also been demonstrated in breast cancers [43]. The immunosuppressive subset of CAFs (CAF-S1) described by Costa and collaborators was shown to recruit T lymphocytes and induce their differentiation towards CD25 antigen (CD25) ^High^ forkhead box P3 (FOXP3) ^High^, which suggestively explains the accumulation of FOXP3^+^ T lymphocytes in some triple negative breast cancers. Subpopulations of CAFs distinguished by scRNA-seq were also described by Li *et al*. in colorectal cancer [44]. In this context, however, CAFs subpopulation were mainly distinguished according to the high expression of ECM remodeling-related genes (CAF-A) or cell motility-related genes (CAF-B). Interestingly, CAFs subpopulations may be characterized not only by different phenotypes, but also according to distinct spatial localization within the tumor mass. For instance, whereas myCAFs have been frequently reported adjacent to and in direct contact with cancer cells, most iCAFs seem to localize more distant from the cancer cells [37, 41].

**Fig. 1:**
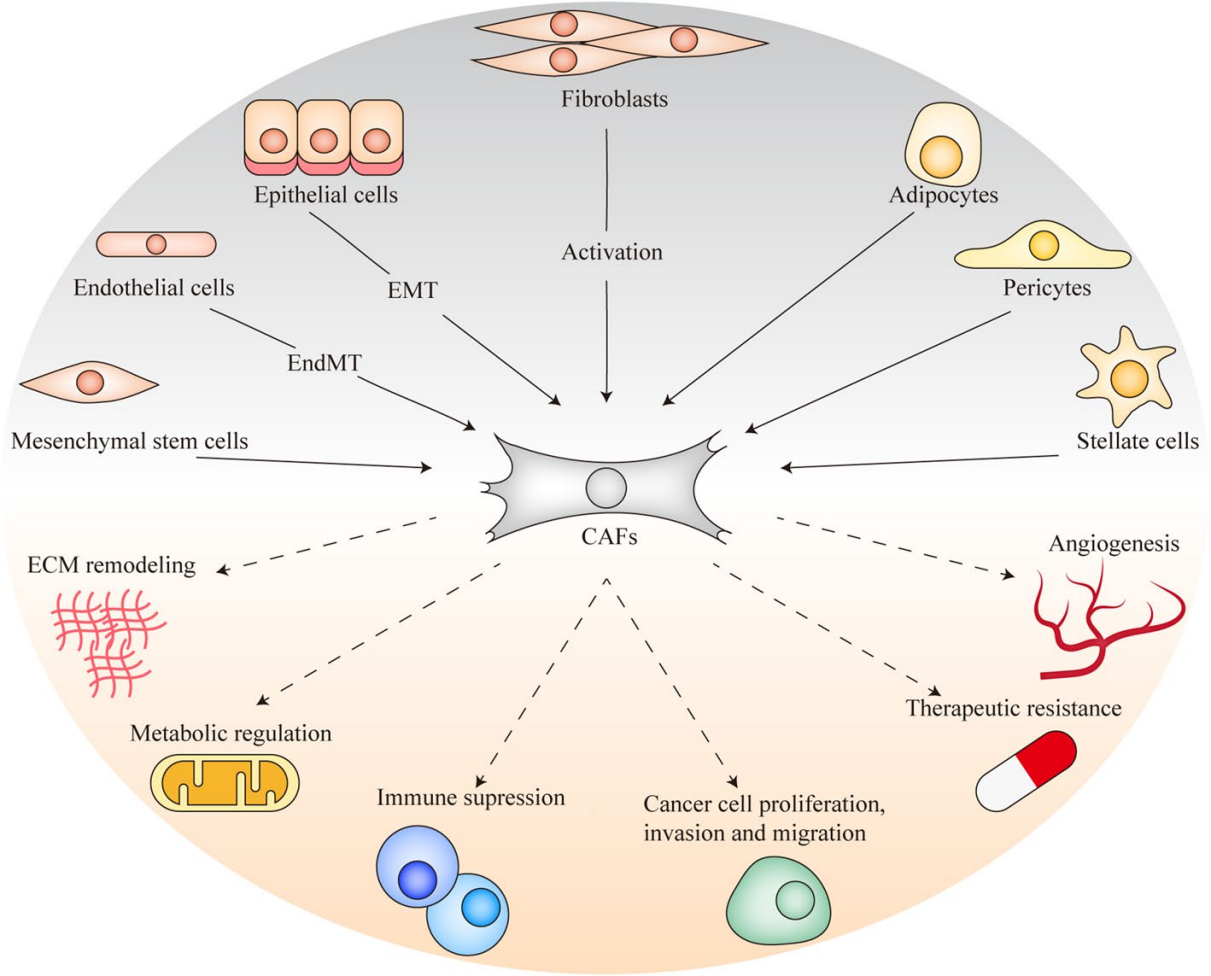
The origins and functions of CAFs in cancer progression. In the TME, CAFs can originate from resident fibroblasts by activation, epithelial cells following EMT, endothelial cells via EndMT, bone marrow-derived mesenchymal stem cells, pericytes, adipocytes and hepatic stellate cells by trans-differentiation. In most cases CAFs have cancer-promoting functions. CAFs play an important role in ECM remodeling by secreting ECM components and enzymes. CAFs can not only serve as physical barriers to protect the cancer cells from the external drugs and the attack of immune cells, but also secrete abundant soluble factors, EVs and ECM to regulate other cells type in TME, which include stimulating cancer cell proliferation, invasion and migration, angiogenesis and therapy resistance. Moreover, CAFs can regulate cancer cell metabolism and stimulate immune evasion of cancer cells

Whereas CAFs localization within the tumor stroma may contribute to the existence of distinct subpopulations (e.g. myCAFs or iCAFs), CAFs differentiation from different progenitors may also account for their heterogeneity. Resident fibroblasts, bone marrow-derived mesenchymal stem cells, epithelial cells following epithelial-to-mesenchymal transition (EMT), endothelial cells via endothelial-to-mesenchymal transition (EndMT), pericytes, adipocytes and other specialized mesenchymal cells such as stellate cells have all been described to originate CAFs [19, 23, 24, 45] (Fig. 1). Promisingly, the emerging scRNA-seq analysis of CAFs in different cancers will provide better biomarkers for the characterization of different subgroups of CAFs with distinct functions, especially for the CAFs with anti-tumor role and pro-tumorigenic role [42, 46–48]. The identification of specific markers for CAFs that act in an opposing manner in cancer progression may lead to improved CAF targeting for treatment of cancer patients.

### The functions of CAFs in cancer development

CAFs can be functionally heterogenous with even opposing roles in cancer development. In most cases CAFs exert cancer-promoting functions, but also in some occasions anti-tumorigenic functions have been described [15, 39]. The heterogeneity of CAFs also provide more challenges for CAFs targeting therapies, which requires the anti-CAFs therapeutic approaches should be more specific to the pro-tumorigenic CAFs [49].

### Anti-tumor roles of CAFs

CAFs may exert tumor suppressor functions when they are activated by epithelial injury in the initial stages of cancer progression [15, 17, 50]. The activated fibroblasts contract the extracellular matrix so that the wound edges remain together, secrete matrix proteins to repair the remaining tissue injuries, and attract epithelial cells to complete the healing process [17]. Also, part of the CAF secretome may also have anti-tumor functions including transforming growth factor-β (TGF-β), which can restrain tumor initiation in the early stage of carcinogenesis [51]. However, with the gradual growth of tumors, this repairing process may in turn switch and promote tumor growth, because cancer cells utilize the growth factors secreted by CAFs, to promote their survival and proliferation [15].

It is also possible that the anti-tumor role described for CAFs is restricted to specific subtypes of CAFs in particular cancer types. Specifically, Meflin was shown as a marker for anti-tumorigenic CAFs in pancreatic ductal adenocarcinoma (PDAC). Infiltration of Meflin-positive CAFs was associated with good prognosis for PDAC patients. In line with this notion, overexpression of Meflin in CAFs inhibited the tumor growth, whereas the loss of Meflin promoted the tumor progression in a PDAC mouse model [39]. Similarly, in another PDAC mouse model, the deletion of α-SMA^+^ CAFs was also shown to promote cancer progression by increasing the number of CD4^+^ Foxp3^+^ regulatory T (Treg) cells in tumors, suggesting an important anti-tumor role for α-SMA^+^ CAFs in PDAC [52]. In estrogen receptor (ER) positive breast cancer, CAFs can be divided into two subtypes with opposite functions based on CD146 expression. The CD146-positive CAFs have been proved to maintain ER expression in ER positive breast cancer cells and remain estrogen responsive and sensitivity to tamoxifen, while CD146-negative CAFs can inhibit the response of cancer cells to tamoxifen and lead to poor treatment outcomes [53]. Besides, the versican (VCAN) is also a potential marker for the tumor-repressing CAFs. The depletion of VCAN in QRsP11 murine fibrosarcoma cells was demonstrated to promote tumor growth and angiogenesis in the mouse model. Specially, the loss of VCAN in QRsP11 cells inhibited collagen biosynthesis and proliferation of fibroblasts, and then reduce the collagen stiffness [54]. This dysregulation of the ECM structure may facilitate the sprouting of endothelial cells and tube formation toward angiogenesis. Based on these studies, the non-selective targeting of whole CAF population maybe not efficient for all cancer types. More reliable and specific markers for anti-tumor CAFs in different cancers remain to be discovered to improve the precision of targeting treatment [55].

### Pro-tumorigenic roles of CAFs

CAFs can promote cancer development in multiple aspects, including stimulating cell proliferation, invasion and migration of cancer cells, angiogenesis and therapy resistance. CAFs can also regulate immunity and metabolism to promote tumorigenesis through secreting cytokines, chemokines, EVs and the ECM [15, 36, 56] (Fig. 1).

Promoting the proliferation, invasion and migration of cancer cells is a major way of CAFs to facilitate the cancer development. Cytokines secreted by CAFs that have been implicated in this process include TGF-β, interleukin-1α (IL-1α), IL-6, interleukin-33 (IL-33), stromal cell-derived factor 1 (SDF1), C-X-C motif chemokine ligand 8 (CXCL8) and cyclooxygenase-2 (COX-2) [15, 57–61], and different molecules mediate diverse effects. For example, secretion of TGF-β by CAFs promotes the EMT of breast cancer cells via TGF-β/SMAD and non-SMAD signaling pathways [62, 63], and facilitates the tumor growth and metastasis in colorectal cancer [64, 65]. It is noteworthy that secretions of certain cytokines by CAFs, such as TGF-β, can cause a positive feedback loop that leads to a prolonged CAF overactivation [37]. Importantly, CAFs also promote the invasion of cancer cells through forming invadopodia and secreting matrix metalloproteinases (MMPs) to degrade the surrounding ECM [56].

In addition, the initiation of tumor vascularization provides an environment that enables rapid tumor growth and facilitates metastasis [66]. Vascular endothelial growth factor (VEGF) is a pivotal factor secreted by CAFs to stimulate new vessel formation, which can be increased by other extracellular cues, such as hypoxia [67]. Other examples of pro-angiogenic factors produced by CAFs are Wnt family member 2 (WNT2), TGF-β, MMPs, fibroblast growth factors (FGFs), angiopoietin-1 and angiopoietin-2 [68, 69].

CAFs play a crucial part in immunosuppression by producing multiple cytokines, such as TGF-β, IL-6, SDF-1, C-X-C motif chemokine ligand 5 (CXCL5) and C-X-C motif chemokine ligand 12 (CXCL12) [36, 70]. Macrophages are the main leukocytes infiltrating solid tumor tissues [71]. CAFs can induce macrophages to polarize from pro-inflammatory M1 into a resolving inflammation M2-like phenotype in pancreatic cancer and prostate cancer [72]. The M2 phenotype is the TAM phenotype promoting EMT and invasion of cancer cells. CAFs-derived IL-6, SDF-1 and macrophage colony-stimulating factor (M-CSF) have been reported to involved in M2 macrophage polarization [73, 74]. Besides, immune cells such as T cells and NK cells can be functionally suppressed by CAFs, by secretion of programmed cell death 1 (PD-1), programmed cell death 2 (PD-2), CXCL5 and prostaglandin E2 (PGE2) [75, 76].

CAFs can modulate the metabolism of cancer cell in multiple ways. Cancer cells were found to undergo metabolic reprogramming and increase aerobic glycolysis to produce ATP even in normal oxygen levels, which was known as the Warburg effect [77, 78]. The similar phenomenon can also happen in CAFs [79]. The CAFs undergo aerobic glycolysis and secret energy metabolites including lactate and pyruvate to the adjacent cancer cells, which enhance the ATP production and increase cancer cell proliferation [79–81]. This metabolic symbiosis phenomenon is termed as Reverse Warburg effect. Furthermore, the loss of caveolin-1 (Cav-1) in CAFs can increase the expression of glycolytic enzymes and promote tumor growth and angiogenesis, which has been proposed as biomarker for the Reverse Warburg effect [79, 82, 83]. In addition, ECM components produced by CAFs and CAFs-derived cytokines including C-C motif chemokine ligand 5 (CCL5), IL6, and C-X-C motif chemokine ligand 10 (CXCL10) were found to regulate cancer cell metabolism by impacting on different signaling pathways [84–86].

The therapy resistance of tumors is also linked to CAF activation. CAFs not only provide a physical barrier by increased interstitial fluid pressure that impedes therapeutic drugs from reaching cancer cells, but also by secreting various proteins and cytokines that can attenuate the efficiency of chemotherapy. For example, SDF-1 secreted by CAFs triggered malignant progression and gemcitabine resistance in pancreatic cancer by enhancing the expression of special AT-rich sequence-binding protein-1 (SATB-1) in cancer cells [57].

## The communication between CAFs and other cells

**Fig. 2:**
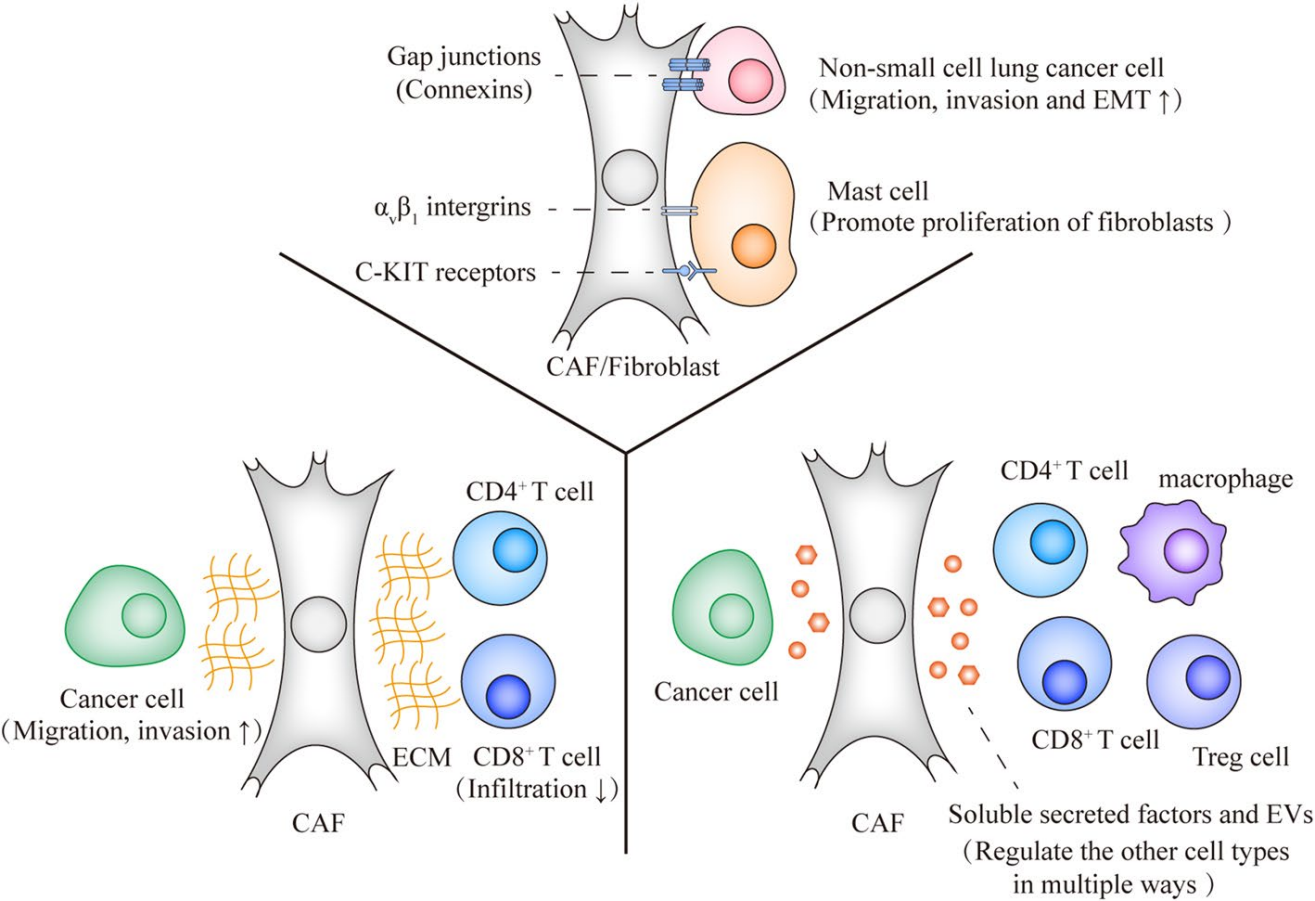
The communication ways between CAFs and other cells. Examples are depicted of the different manners of cross-talk between CAFs and other cell types; they can be mainly divided into three groups: (i) cell-to-cell junctions, (ii) ECM interactions and (iii) the interactions mediated by secreted cytokines, growth factors, chemokines, peptides, amino acids and EVs, including exosomes

As mentioned before, the interplay between CAFs and other cells favors cancer progression by mediating, for example, cancer cell proliferation, invasion, and immune evasion. Within the TME, this interplay occurs via multiple different modes of action. Cross-talk between CAFs and other cells can be mediated by direct cell-to-cell contact or indirect communication. The direct cell-to-cell contact can involve gap junctions, tunneling nanotubes, direct transmembrane ligands interacting with transmembrane receptors, and adhesion molecules. Conversely, the indirect interaction relies on secreted cytokines, growth factors, chemokines, peptides, amino acids and EVs [87, 88], which can act over short or long distances. Moreover, CAFs efficiently change the ECM constitution. Further interaction between cell surface proteins and ECM molecules plays an important role on cancer progression and therefore might also be considered an important mechanism for the crosstalk between CAFs and other cell types within the TME (Fig. 2).

Although different strategies greatly contribute for the crosstalk between distinct cell types within a tumor, the next sections dedicate a special attention to the indirect interaction between CAFs and other cells. Among the mechanisms that may be used in this process, an interesting part has recently emerged for exosomes and their role is then discussed as critical mediators of cargo transference from CAFs to other cells within the tumor stroma.

### Cell-cell junctions

Several mechanisms enable the transference of cellular cargos from CAFs to cancer cells and *vice versa*. Cell-cell junctions play an important role mediating the interaction between these cells upon direct cell contact. For example, the intercellular space of gap junctions is between 2 and 4 nm, while tunneling nanotubes are 50–100 μm in length [89, 90]. Specifically, gap junctions that consist of six connexins, mediate gap junctional intercellular communication (GJIC) by physical contacts between CAFs and other cells. They are able to mediate the transfer of rather small molecules and metabolites that are up to 1000 Da [91]. For instance, in non-small cell lung cancer (NSCLC), connexin 43 (Cx43)-formed unidirectional GJIC was found to play an important role in regulating metabolic cooperation between NSCLC cells and CAFs. CAFs undergo aerobic glycolysis (i.e., the Reverse Warburg effect) in TME as mentioned before. CAFs undergoing aerobic glycolysis can enhance the OXPHOS of NSCLC cells by transferring tricarboxylic acid (TCA) metabolites, including ATP, to NSCLC cells through Cx43-formed unidirectional GJIC. Therefore, this process can activate the phosphatidylinositol 3-kinase/protein kinase B (PI3K/AKT) and mitogen-activated protein kinases/extracellular signal-regulated kinase (MAPK/ERK) signaling pathways in NSCLC cells. These effects promote migration, invasion and EMT of NSCLC cells [92]. Similarly, adjacent cells can communicate with each other via contact-dependent signaling like ligand-receptor pairs and other ways of cell adhesion. For example, α_v_β_1_ integrins and c-KIT receptors on the cells surface of HMC-1 mast cells can mediate the attachment to fibroblasts in chronically inflamed tissues [93, 94]. The release of interleukin-4 (IL-4) by mast cells induced by heterotypic cell-cell adhesion between mast cells and fibroblasts is an important stimulation of fibroblast proliferation [93, 95, 96].

### ECM interactions

CAFs can indirectly affect cancer cell behavior and other cell types in the TME by secreting ECM proteins and remodeling the ECM. ECM proteins including integrins, matricellular proteins, structural ECM proteins (collagen, fibronectin) and metalloproteinases that can serve as signaling mediators between fibroblasts and cancer cells [97]. The production of these ECM components can remodel the ECM and affect their organization, including ECM stretching, crosslinking, aligning, bundling, and stiffening [56]. CAFs can also degrade the ECM through secreting specific proteases such as MMPs [98]. Certain ECM modifications, such as high contractility, are a feature when fibroblasts transform into CAFs. CAFs can remodel the ECM and create the paths for cancer cells to migrate [99], and thereby facilitate cancer cell invasion. For example, FAP was shown to be highly expressed in CAFs rather than cancer cells and normal tissue in pancreatic cancer. The overexpression of FAP in CAFs can organize fibronectin and collagen I fibers into parallel orientation, which can elevate directionality and velocity of cancer cells in the ECM [100]. Besides, ECM also play a crucial role in the regulation of immune cell trafficking. For instance, the decrease of hyaluronan and proteoglycan link protein 1 (HAPLN1) in aged fibroblasts was shown to lead to less contractile ECM that impeded the infiltration CD4^+^ and CD8^+^ T cells, and promoted metastasis of melanoma cells [101].

### Soluble secreted factors

CAFs are a substantial source of cytokines, chemokines, growth factors and other secreted factors in the TME. One of the most studied CAFs-secreted cytokines is TGF-β, whose pathway is crucial in promoting tumor progression in various cancer subtypes [34]. TGF-β and other growth factors and chemokines released by CAFs also act on different types of immune cells including CD8^+^ T cells, Treg cells and macrophages with mostly immune-suppressive consequences [36, 102]. Besides, the metabolites and amino acids secreted by CAFs is another way that mediate the communication between stromal fibroblasts, tumor cells and other cell types in TME [86, 103, 104]. For instance, through the provision of alanine, CAFs further enhance carcinogenesis by allowing cancer cells to fuel the TCA cycle, support lipid and non-essential amino acids (NEAA) synthesis, as well as diverting glucose metabolism to serine and glycine synthesis, both of which are essential for cancer cell survival [105].

### Extracellular vesicles

Besides releasing soluble factors, secreting EVs is a critical determinant to enable autocrine and paracrine signals that promote cancer cell aggressiveness and therapy resistance. The classification of EVs has been updated during recent years and new insights continue to be obtained. According to the Minimal information for studies of extracellular vesicles 2018 (MISEV2018) guidelines, as proposed by the International Society for Extracellular Vesicles (ISEV), the EVs are classified into different subtypes according to the physical characteristics. For example, the size is a commonly used standard to distinguish different subtypes of EVs. The diameter of small EVs (sEVs) is below 100 nm or 200 nm, while the diameter of medium/large EVs (m/lEVs) is more than 200 nm. EVs can also be classified based on other physical characteristics, including density (low, middle, high, with ranges defined), biochemical composition with specific makers such as CD63^+^/CD81^+^ EVs, and origins or conditions such as apoptotic bodies [106]. Historically, exosomes is another widely used definition for a specific subpopulation of sEVs, which range from 30-150 nm and originate from endosomes [27, 28]; vesicles with these properties, termed exosomes have been indicated to play a critical part in physiological and pathological processes [107–109]. In the TME, CAFs are a notable source of exosomes [110].

**Fig. 3:**
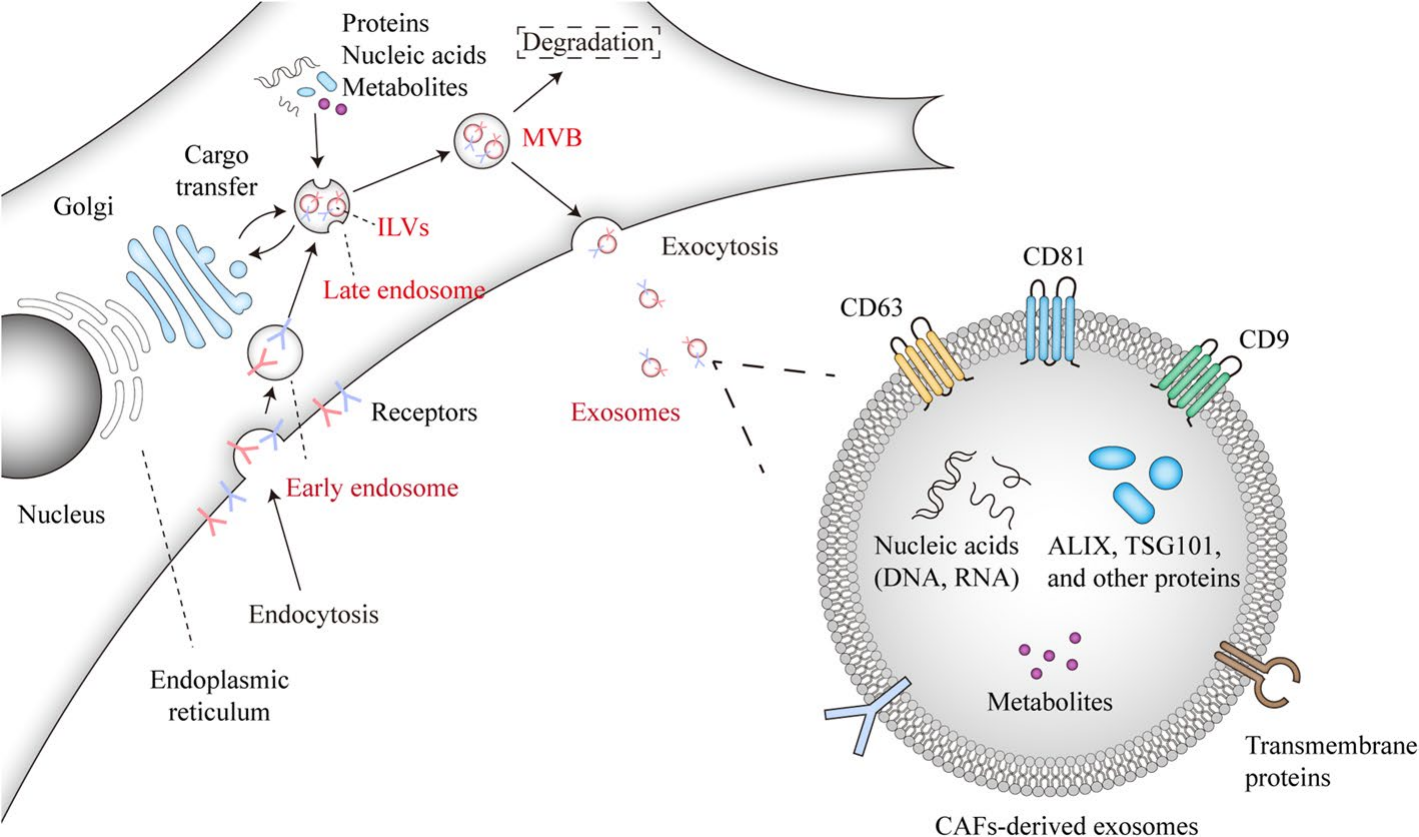
Schematic presentation of exosomes in the TME. The biogenesis of exosomes mainly comprises three stages, which includes (i) the formation of early endosome by cytoplasmic membrane invagination, (ii) the formation of MVBs containing cargos-enriched ILVs, and (iii) the release of ILVs as exosomes after the fusion of MVBs with the plasma membrane

Exosome biogenesis mainly comprises three different stages, including (a) the formation of endocytic vesicles via invagination of the plasma membrane called early endosome; (b) the formation of multi-vesicular bodies (MVBs) containing intraluminal vesicles (ILVs) generated by inward budding of the endosomal membrane with the cytoplasmic constituents; and (c) the fusion of MVBs with the plasma membrane and extracellular release of ILVs as exosomes [28, 111, 112] (Fig. 3). Also, the MVBs can be degraded through fusing with lysosomes or autophagosomes [113]. A multitude of proteins are involved in the maturation of MVBs and ILVs, including endosomal sorting complex required for transport (ESCRT) proteins which consist of four different protein complexes, ESCRT-0, −I, −II, and -III [28, 114, 115]. In addition to ESCRT proteins, apoptosis-linked gene 2-interacting protein X (ALIX) [116], vesicle trafficking 1 (VTA1) [117], soluble N-ethylmaleimide-sensitive factor attachment protein receptor (SNARE) [118] and GTPases are also considered as important players in exosome biogenesis and secretion [119].

After secretion, exosomes are taken up by neighboring cells via endocytosis or by fusion with the plasma membrane [120, 121]. After internalization, exosomes can be degraded by lysosomes in recipient cells. Also, the internalized exosomes may fuse with endosomes, and then disintegrate and release vesicle contents into the cytoplasm or fuse back with the plasma membrane and release exosomes to the outside of recipient cells [113, 122–124].

Exosomes are highly heterogeneous, which is reflected in differences in their sizes and contents [113]. With respect the later, cellular origin and signaling from extracellular environment have great impact on heterogeneity. Exosomes are composed of metabolites, lipids, functional proteins including membrane proteins, cytosolic and nuclear proteins, extracellular matrix proteins, and nucleic acids including mRNAs, noncoding RNAs, and DNA fragments [113, 125, 126]. The contents of exosomes are important regulators of cellular functions and pathological states, such as tumorigenesis, immune responses, inflammatory reactions and cell death [113]. In TME, CAFs-derived exosomes also regulate tumor growth, metastasis, angiogenesis, and mediate therapy resistance of tumor cells [110].

## The role of exosomes secreted by CAFs on other cells in the TME

### The impact of CAFs-derived exosomes on cancer cells

The secretion of exosomes is an important way of CAFs to influence cancer cell behavior [110, 127]. Consistent with this notion, CAFs-derived exosomes have been associated with several hallmarks of cancer. The abnormal expression of molecules contained in the CAFs-derived exosomes can lead to the dysregulation of signaling pathways in cancer cells after the uptake of exosomes by cancer cells (Table 1). Also, these dysregulated molecules including non-coding RNAs in the CAFs-derived exosomes are potential biomarkers for the specific cancer types.

**Table 1:** The role of cargos in CAFs-derived exosomes on regulating the cancer cells

Cancer type	Molecule in exosomes	Expression	Mechanism	Impact on cancer cells	Ref
Bladder cancer	miR-148b-3p	Up-regulated	Target PTEN and activate Wnt/β-catenin pathway	Promote tumor proliferation, metastasis and drug resistance	[128]
Breast cancer	miR-181d-5p	Up-regulated	Target CDX2 and downregulate CDX2 and HOXA5	Enhance the aggressiveness of breast cancer	[129]
	SNHG3 (lncRNA)	Up-regulated	Target miR-330-5p and increase the PKM expression	Increase glycolysis metabolism	[130]
	miR-21, miR-378e, miR-143	Up-regulated	Not mentioned	Induce of the stemness and EMT phenotype of breast cancer	[131]
	ADAM10	Up-regulated	Activate RhoA and Notch signaling	Promote cells motility and tumor progression	[132]
	miR-500a-5p	Up-regulated	Target USP28 and downregulate USP28	Promoted the proliferation and metastasis of breast cancer cells	[133]
	miR-22	Up-regulated	Target ESR1 and PTEN, and downregulate ESR1 and PTEN.	Promote tamoxifen resistance	[134]
	circHIF1A	Up-regulated	Increase the expression of CD44 by targeting and downregulating miR-580-5p	Promote breast cancer cells proliferation and stemness in hypoxic stress	[135]
(Triple-negative breast cancer)	miR-4516	Down-regulated	Target FOS like antigen 1 (FOSL1)	Promote the development of TNBC	[136]
Colorectal cancer	H19 (lncRNA)	Up-regulated	Activate the β-catenin pathway	Promote the stemness of cancer stem cells	[137]
	LINC00659	Up-regulated	Target miR-342-3p and downregulate miR-342-3	Promote cancer cells proliferation, invasion, migration and EMT progression	[138]
	miR-590-3p	Up-regulated	Target CLCA4 and downregulate CLCA4	Promote radiotherapeutic resistance	[139]
	circSLC7A6	Up-regulated	Increase the expression of C-X-C motif chemokine receptor 5 (CXCR5)	Promote cancer cells proliferation and metastasis	[140]
	circEIF3K	Up-regulated	Increase the expression of programmed death-ligand 1 (PD-L1) by targeting and downregulating miR-214	Promote hypoxia-induced CRC progression	[141]
Endometrial cancer	miR-148b	Down-regulated	Target DNMT1 and downregulate DNMT1	Promote cancer cells metastasis by inducing EMT	[142]
	miR-320a	Down-regulated	Target HIF1α and downregulate HIF1α	Promote cancer cells proliferation	[143]
Esophageal cancer	miR-33, miR-326	Up-regulated	Not mentioned	Promote CAF phenotype and tumor progression	[144]
	SHH	Up-regulated	Activate SHH signaling pathway	Improve the growth and migration abilities	[145]
Gastric cancer	miR-522	Up-regulated (Conditional)	Target arachidonate lipoxygenase 15 (ALOX15) and downregulate ALOX15	Inhibit ferroptosis in cancer cells	[146]
	circ_0088300	Up-regulated	Enhance janus kinase 1/ signal transducer and activator of transcription 1 (JAK1/STAT1) signaling pathway by targeting miR-1305 and downregulating miR-1305	Promote cancer cells proliferation, migration and invasion	[147]
Head and neck cancer	miR-3188	Down-regulated	Target BCL2 apoptosis regulator (BCL2) and downregulate BCL2	Promote cancer cells growth	[148]
Hepatocellular carcinoma	miR-320a	Down-regulated	Target PBX homeobox 3 (PBX3) and downregulate PBX3	Promote cancer cells proliferation and metastasis	[149]
Lung cancer	SNAI1	Up-regulated	Not mentioned	Promote EMT in cancer cells	[150]
Oral squamous cell carcinoma	miR-34a-5p	Down-regulated	Target AXL receptor tyrosine kinase (AXL) and downregulate AXL	Promote cancer cells proliferation and metastasis	[151]
	miR-382-5p	Up-regulated	Not mentioned	Promote cancer cells migration and invasion	[152]
	miR-21-5p	Up-regulated	Enhance PI3K/mTOR/STAT3 Signaling	Promote normal gingival fibroblasts (NGFs) to CAFs	[153]
Oral tongue squamous cell carcinoma	MFAP5	Up-regulated	Activate MAPK and AKT pathways	Activate cancer cells growth and migration	[154]
Osteosarcoma	miR-1228	Up-regulated	Target suppressor of cancer cell invasion (SCAI) and downregulate SCAI	Promote osteosarcoma invasion and migration	[155]
Ovarian cancer	miR-98-5p	Up-regulated	Target cyclin dependent kinase inhibitor 1A (CDKN1A) and downregulate CDKN1A	Promote cisplatin resistance	[156]
	TGF-β	Up-regulated	Activate the SMAD signaling pathway	Promote migration and invasion ability of cancer cells and EMT	[157]
Prostate cancer	miR-423-5p	Up-regulated	Target GREM2 and downregulate GREM2	Promote chemotherapy resistance	[158]

### Proliferation, migration and invasion

Although the role of CAFs-derived exosomes in cancer development is likely dynamic and specific to cancer type, genetics and stage, the CAFs-derived exosomes can affect the proliferation, migration and invasion of cancer cells. Exosomal microRNAs (miRNAs) were shown to have important functions in the crosstalk between CAFs and cancer cells. Numerous CAF-derived exosomal miRNAs have been identified and shown to play a pivotal role in cancer development. For examples, miR-181d-5p, miR-500a-5p, miR-21, miR-22, miR-378e and miR-143 were found to be upregulated in breast CAFs-derived exosomes compared exosomes secreted by normal fibroblasts [129, 131, 133, 134]. In breast cancer, CAFs-derived exosomes carrying miR-181d-5p can promote proliferation, invasion, migration, and EMT and inhibit apoptosis of cancer cells by targeting caudal-related homeobox 2 (CDX2) and then downregulating CDX2 and its downstream gene -homeobox A5 (HOXA5) [129]. Besides, the exosomal miR-500a-5p can promote breast cancer cell proliferation and metastasis by targeting and reducing the expression of ubiquitin-specific peptidase 28 (USP28) [133]. In addition, some circular RNAs (circRNAs) were also found to have tumor-promoting roles in CAFs-derived exosomes. Specifically, the level of exosomal circHIF1A derived from hypoxic CAFs was increased compared to the normoxic CAFs in breast cancer. The circHIF1A can act as a sponge for miR-580-5p and decrease the level of miR-580-5p, which was shown to target the CD44 molecule (CD44) mRNA and decrease the expression of CD44. In this way, the up-regulated circHIF1A from CAFs-derived exosome in hypoxia can boost the expression of CD44 indirectly, and then enhance the cancer stem cell plasticity in TME [135]. In colorectal cancer, CAF-secreted exosomal circEIF3K was shown to inhibit cancer cell proliferation, invasion and tube formation *in vitro*, suggestively by targeting miR-214 and impairing its activity [141]. Similar to miRNAs and circRNAs, the secretion of long noncoding RNAs (lncRNAs), via CAFs exosomes have been proved to have cancer-promoting abilities. In colorectal cancer, for example, LINC00659 can be delivered from CAFs to cancer cells via exosomes. The up-regulated LINC00659 interacts directly with miR-342-3p, and increases Annexin A2 (ANXA2) expression in CRC cells. The latter promotes CRC cell proliferation, migration, invasion and EMT progression *in vitro* [138]. On the other hand, many miRNAs that are down-regulated in CAFs-derived exosomes compared with normal fibroblasts-derived exosomes also have the link with cancer progression. MiR-148b and miR-320a are expressed at lower levels in endometrial cancer cells and CAFs compared to normal cells, and their expression is also reduced in CAFs-derived exosomes compared to normal fibroblasts-derived exosomes. The miR-148b and miR-320a target DNA methyltransferase 1 (DNMT1) and hypoxia inducible factor 1 subunit alpha (HIF1α), respectively, which have the potential of promoting cancer cell metastasis and angiogenesis [159, 160]. Therefore, whereas exosomes secreted by normal fibroblasts might impair cancer progression by downregulating DNMT1 and HIF1α in cancer cells through delivering miR-148b and miR-320a, the lack of inhibition by CAFs-derived exosomes could boost the cancer progression [142, 143].

The transfer of exosomal proteins from CAFs to cancer cells can also promote tumorigenesis. The breast CAFs derived-exosomes, which contained highly expressed ADAM metallopeptidase domain 10 (ADAM10), promoted cell motility by activating RhoA signaling in breast cancer cells [132]. Meanwhile, ADAM10 plays an important role in the activation of Notch signaling cascade through promoting Site-2 cleavage of the Notch receptor, followed by γ-secretase-mediated Site-3 cleavage to generate Notch intracellular domain and initiate signalling [161]. In oral tongue squamous cell carcinoma (OTSCC), microfibril associated protein 5 (MFAP5) was demonstrated to be enriched in CAFs derived-exosomes. The exosomal transfer of this protein was found to promote OTSCC cell growth and migration by inducing the activation of MAPK and AKT pathways [154]. Also, Sonic Hedgehog (SHH) was detected at highl levels in CAFs-derived exosomes. The exosomes mediated transfer of SHH from CAFs to cancer cells improved proliferation and migration abilities of the esophageal cancer cells in esophageal squamous cell carcinoma (ESCC) [145].

### Metabolism

The CAFs-derived exosomes also have a crucial role in inducing metabolic reprogramming in cancer cells, which is a hallmark of cancer development. The CAFs-derived exosomes of prostate cancer, pancreatic cancer and breast cancer have the function in downregulating the mitochondrial function of the cancer cells by inhibiting oxygen consumption rate (OCR) [130, 162]. In prostate cancer, several miRNAs contained in CAFs-derived exosomes were found to have the ability to downregulate mitochondrial oxidative phosphorylation and reprogram metabolic pathways in cancer cells, such as mir-22, let7a and mir-125b [162]. In breast cancer, the CAFs-derived exosomal small nucleolar RNA host gene 3 (SNHG3), which is a lncRNA that acts as a molecular sponge of miR-330-5p to up-regulate pyruvate kinase M1/M2 (PKM) expression, can lead the inhibition of mitochondrial oxidative phosphorylation and enhanced breast tumor cell proliferation [130]. Moreover, the inhibition of mitochondrial oxidative phosphorylation by CAFs-derived exosomes is associated with a compensatory increase in glycolysis (i.e., the Warburg effect) [130, 162]. Specially, the exosomes can decrease the percentage conversion of glucose to α-ketoglutarate and instead divert it towards lactate in cancer cells.

Furthermore, the CAFs-derived exosomes can increase the level of glutamine for biosynthesis in prostate cancer cells and pancreatic cancer cells by switching the carbon source from the oxidative glucose pathway towards glutamine via the reductive carboxylation pathway in the TCA cycle [162]. In addition, the CAFs-derived exosomes also act as a source of metabolite cargos, TCA cycle metabolites, amino acids, and lipids, which can fuel the metabolic activity of the prostate and pancreatic cancer cells [162].

### Therapy resistance

Therapy resistance is a frequent cause of tumor recurrence and treatment failure of cancer patients [163, 164]. Uptake of CAFs-derived exosomes by cancer cells has been linked to this response.

Tamoxifen is the most commonly used drug for the treatment of ER positive breast cancer [165]. However, many breast cancer patients eventually develop tamoxifen resistance and show a poor prognosis [134]. The tamoxifen resistance in cancer cells can be caused by multiple mechanisms, such as the dysregulation of the ER signaling pathway and PI3K/AKT pathway [166]. Recently, the CD63^+^ CAF-derived exosomes have been confirmed to perform a crucial role in mediating tamoxifen resistance. The miR-22 is highly expressed in CD63^+^ CAF-derived exosomes, which can target estrogen receptor 1 (ESR1) and phosphatase and tensin homolog (PTEN), and suppress the expression of ESR1 and PTEN [134]. The loss of PTEN promotes tamoxifen resistance in breast cancer [134, 167].

The CAFs-derived exosomes can also promote the chemotherapy resistance of the cancer cells. Specifically, miR-423-5p transported by the CAFs-derived exosomes can promote resistance to taxane by targeting gremlin 2 (GREM2) and promoting TGF-β signaling in prostate cancer. The cancer cells with increased level of miR-423-5p exhibited elevated cell proliferation and the reduced cell apoptosis rate when exposed to taxane [158].

Radiotherapy is being increasingly used as a preoperative treatment owning to its efficiency and effectiveness in lessening the local recurrence of advanced cancer [168]. However, patients often develop resistance to radiotherapy and convert to more aggressive phenotypes, which is partially due to the heterogenous components in TME. In CRC, the CAFs-derived exosomes have been reported as an important cause of radio resistance of cancer cells by preventing the DNA damage and inhibiting apoptosis and in CRC cells. These results were associated with reduced activities of cleaved-caspase 3 and cleaved-poly (ADP-Ribose) polymerase 1 (PARP) induced by CAFs-derived exosomes [139]. The up-regulated miR-590-3p in CAFs-derived exosomes compared with normal fibroblast-derived exosomes was confirmed to play a critical role in this regulation by targeting at chloride channel accessory 4 (CLCA4) and activating the PI3K/AKT signaling pathway [139].

### The impact of CAFs-derived exosomes on immune function

As discussed in the previous section, CAFs-exosomes can contribute to cancer progression through different mechanisms. Their uptake by cancer cells can (de) activate signaling pathways crucially associated with cell proliferation, migration and invasion [107]. Still, non-cancer cell types are also expected to interact with and uptake exosomes secreted at the TME, and the impact of exosomes on these cells may also be critically involved with cancer progression by regulating distinct cancer hallmarks. CAFs have been shown to induce angiogenesis, inflammation and immune suppression, and their impact on the progression of malignant lesions were reviewed by others [23, 37]. Still, the specific contribution of CAFs-exosomes to these process remains largely unexplored. Therefore, this section presents the initial data available in the literature that reports how CAFs-derived exosomes can impact immune cells, and discusses it by considering known effects triggered by cancer cell-secreted exosomes mediating immune suppression.

Antitumor immunity is a natural defense against cancer which is in part mediated by immune cells such as CD8^+^ T cells and NK cells [169]. Specifically, the cytotoxic CD8^+^ T cells are important for immune-mediated tumor elimination [170, 171]. In order to exert their cytotoxic effect the T cells needs to complete several steps including T cell activation, expansion, differentiation and infiltration [34]. This antitumor response can be regulated by multiple cell types, including Treg cells, myeloid cells, endothelial cells, fibroblasts and tumor cells [34].

However, the antitumor response of immune system can meet impediments that contribute to cancer progression. For example, the up-regulated expression of PD-L1 on the surface of cancer cells improve the ability of evading the immune surveillance of cancer cells, which interacts with PD-1 on T cells and mitigate the immune checkpoint response [172]. In melanoma, lung cancer and breast cancer, the cancer cells-derived exosomes have been found to mediate immune suppression via exosomal PD-L1 that inhibit CD8^+^ T cell functions and promote cancer development [173]. The PD-L1 was found to be carried by exosomes on their surface, and this can be up-regulated by interferon-γ (IFN-γ). Importantly, the level of PD-L1 in exosomes isolated from plasma of melanoma patients is remarkably higher than that in healthy donors, which positively correlated with metastasis in melanoma patients [173].

Recently, the breast CAFs-derived exosomes have also been found to have inhibitory effect on antitumor immunity. In breast cancer, the PD-L1 expression of cancer cells can be up-regulated after the uptake of CAFs-derived exosomes. Specifically, miR-92 was found to be enriched in CAFs-derived exosomes. After being absorbed by cancer cells, the miR-92 targets large tumor suppressor kinase 2 (LATS2) that interacts with Yes-associated protein 1 (YAP1) and regulates nuclear translocation of YAP1 in breast cancer cells. After the nuclear translocation, YAP1 can bind to the enhancer region of PD-L1 and promote transcription activity, which increases the level of PD-L1 in cancer cells [174]. The increased PD-L1 significantly induced apoptosis and impaired proliferation of T cells and also blocked the cell-killing function of NK cells [174].

## The clinical impact of exosomes

### The multicomponent biomarker role in cancer biopsy

Cancer is a complex and heterogeneous disease that is in a dynamic flux and subject to interplay with host response [175]. Next to histological analysis, detection of cancer biomarkers is an important way to determine the cancer status. Liquid biopsy-based biomarkers for cancer are gaining significance to improve early detection, help diagnosis, predict prognosis and monitor treatment response [26].

The large number of exosomes circulating in body fluids, with unique biogenesis and ubiquitous production by all cell types, are emerging to be a crucial component for biomarker discovery in liquid biopsies. The exosome contents include nucleic acids, lipids, and proteins that can be developed in potential cancer biomarkers. Such biomarkers can inform about the abnormal cancer signaling, the stromal response of tumors, and the physiological status of secretory cells [26]. In addition, there are a considerable proportion of stromal cell-derived exosomes contained in liquid biopsies including CAFs-, immune cells-, mesenchymal cells-, epithelial cells- and endothelial cells-derived exosomes, which could also reflect the host response to cancer pathology.

Recently, a large-scale and comprehensive proteomic analysis of exosomes and exomeres (nonvesicular particles <50 nm) from 426 human samples was performed to identify universal exosome markers to improve the cancer detection [27, 176]. In this research, the authors defined small exosomes, large exosomes and exomeres collectively as extracellular vesicles and particles (EVPs). Firstly, the proteins composition of EVPs was implicated to be different in various cancer types including pancreatic adenocarcinoma (PaCa) and lung adenocarcinoma (LuCa). In EVPs of PaCa, proteins related with actin cytoskeletal-linked signaling pathways, EMT and coagulation were abundant, while the proteins involved in RNA processing pathways, cell cycle and metabolism were enriched in EVPs of LuCa. Although there are several shared tumor specific EVP proteins that were also enriched in tumor tissues of PaCa and LuCa, most highly enriched EVP proteins were found to be specific to each respective tumor type. This study demonstrated the heterogeneity of different tumor types at EVPs level. Furthermore, the immunoglobulin-related proteins were enriched in plasma-derived EVPs, which could be an efficient type of biomarkers to distinguish normal and cancer samples. Also, the tumor-associated EVP proteins signature in plasma could be detected before the early stage of metastases. Thus, plasma-circulating EVP proteins could be potential biomarkers for early cancer detection [27].

Notably, the exosomal PD-L1 is a potential maker of immunotherapy treatment response, due to the negative correlation between exosomal PD-L1 levels in plasma of cancer patients and the rate of response to anti-PD-1/PD-L1 therapy and patients’ survival [177–179]. Recently, there is some research indicating that the depletion of exosomal PD-L1 can improve the efficiency of anti-PD-L1 blockade in animal models [180, 181]. However, the mechanism of the exosomal PD-L1 in inhibiting the efficiency of anti-PD-1/PD-L1 therapy remains largely unknown. The possible mechanism could be the interaction between circulating exosomal PD-L1 and the anti-PD-1/PD-L1 antibodies, which causes fewer effective antibodies to exert their function in the TME [177]. There are also evidences demonstrated that the elimination of exosomes by inhibiting generation and secretion or extracorporeal hemofiltration may act as an effective add-on therapy to enhance the efficiency of anti-PD-1/PD-L1 therapy [180, 182, 183], which may provide new clinical application prospects for cancer treatment in future [184].

Up to now exosomes and other EVs have been used as biomarkers for early diagnosis of cancer in many clinical trials. For example, in an ongoing clinical trial, researchers collected blood plasma samples from 420 lung cancer patients and 150 healthy controls to purify the exosomes. Deep-learning analysis of exosomes will be used to screen protein makers to distinguish between healthy controls and lung cancer patients, and then distinguish the early stages of lung cancer patients to improve the survival rates (ClinicalTrials.gov Identifier: NCT04529915). Although there are still challenges using exosomes as a source of biomarkers, exosomes still hold a promising and powerful place in liquid biopsy of cancer [26].

### Exosome therapeutics

Exosomes have a promising prospect of therapeutic applications, as they have the ability of intercellular communication and carrying a variety of cargo molecules. Exosomes can serve as delivery vehicles. They can be loaded with therapeutic proteins, therapeutic genes, chemotherapeutic drugs, and small molecules to change the behaviors of target cells [185]. Exosomes can protect the cargos from degradation or neutralization in the body [185, 186]. The biocompatibility of exosomes, with appropriate modifications, can improve the stability and effectiveness of therapeutics, and enhance the absorption by target cells [187].

As efficient drug carriers, the exosomes have potential to be used for cancer immune therapy. For example, they can enable more efficient anti-cancer vaccines development [187]. Specifically, exosomes can be utilized as the vehicles for carrying protein antagonists in cancer-immune regulation. The CD47 overexpressed on the surface of tumor cells can limit the ability of macrophages to engulf tumor cells by interacting with signal regulatory protein α (SIRPα) on macrophages; SIRPα acts as a “don’t eat me” signal. The exosomes carrying SIRPα variants can antagonize the interaction between CD47 and SIRPα and promote the phagocytosis by macrophages. Also, the SIRPα-enriched exosomes can promote the T cell infiltration in the mouse model, and inhibit the tumor growth ultimately [188]. Furthermore, the native exosomes can be engineered to provide more efficient therapy effects. For example, the pH-responsive exosome nano-bioconjugates composed of M1 macrophages-derived exosomes with antibodies of anti-CD47 and anti-SIRPα through acid-cleavable benzoic-imine bonds has been developed to target the tumor cells. These exosome nano-bioconjugates can release the antibodies after the selective cleavage in the acid tumor microenvironment that block the interaction between SIRPα and CD47, and thereby improve phagocytosis of macrophages [189]. For therapy based on CD47 immune checkpoint blockade, another kind of CD47-overexpressed exosomes fused with drug-loaded thermosensitive liposomes was developed. These hybrid nanovesicles, termed hGLVs, can also improve the macrophages-mediated the phagocytosis of tumor cells by blocking CD47 signal, and induce immunogenic cell death during photothermal therapy [190].

Exosomes have also been used in clinical trials. In a completed clinical trial enrolled 41 participants, dendritic cell-derived exosomes have been used to deliver proteins including melanoma antigen recognized by T cells 1 (MART-1) and MAGE family member A1 (MAGEA1) into NSCLC patients, which can suppress progression of lung cancer by activating the immune response with the induction chemotherapy (ClinicalTrials.gov Identifier: NCT01159288). This strategy of tumor vaccination improved the patient survival in phase II clinical trial [191].

The potential of exosomes as therapeutic tools is a promising and rapidly developing research field, which has great potential to improve the prognosis of patients with metastatic cancer. However, there are still many challenges to be overcome in order to achieve better clinical outcomes.

## Concluding remarks

CAFs are a dominant cell type in the TME, which regulates tumor development in various aspects [35]. The heterogeneity of CAFs lead the tumor to be also more heterogeneous and complex. These characteristics contribute to inefficiency of current cancer chemo-, radio-, targeted- and immune therapies [15]. Not only can CAFs act as a physical barrier to protect the cancer cell from drugs and immune cell, but also the abundant secretion by CAFs can feed the cancer cells continuously and limit the efficiency of chemotherapy and function of immune cells [15, 35, 37]. Here, we mainly discussed the role of CAFs-derived exosomes in cancer development. The CAFs-derived exosomes can serve as transport vehicles carrying different cargos from CAFs to other cells within the TME. These cargos including proteins, nucleic acids, metabolites and other molecules that can fuel cancer cells and promote the proliferation, migration and invasion of cancer cells, induce the metabolic reprogramming of cancer cells and promote the therapy resistance of cancer cells. After the uptake of CAFs-derived exosomes, the cancer cells also gain more abilities to escape from the attack of immune cells. The research regarding the impact of CAF-derived exosomes on cancer cells are rapidly emerging. However, the direct role of CAF-derived exosomes on immune cells remains not well understood. The latter is a very interesting and promising area for future research.

As discussed here, CAF-derived exosomes cargo is commonly altered in comparison with normal fibroblasts-derived exosomes. Alterations in the levels of regulatory molecules (e.g. growth factors and non-coding RNAs) can (de) regulate important signaling pathways in recipient cells, therefore contributing to cancer progression. In addition to altered levels of particular cargo, exosomes secreted by cancer cells have been demonstrated to impact recipient cells by transporting mutated proteins with tumor promoting roles, such as KRAS [192]. Conversely, exosomes were also shown to transport proteins coded by tumor suppressor genes with active function in recipient cells, as exemplified by PTEN [193, 194]. The secretion of exosomes carrying non-mutated proteins can also impact mutated cancer cells by reactivating critical molecular pathways. Still, while this possibility could emerge as promising in terms of cancer progression impairment, a cautious interpretation and thorough investigation is otherwise advised before further conclusions. An important example in this matter may involve inactivating mutations in components of the TGF-β signaling pathway, which are frequently observed in some types of cancer (e.g., pancreatic and colorectal cancers). While the TGF-β signaling pathway is known to suppress tumor initiation and promotion by inhibiting cell proliferation and/or inducing apoptosis in normal or early-stage cancer cells, the activation of TGF-β signaling in late-stage tumors promote cancer cell EMT, migration and invasion [34]. Therefore, if non-mutated functional components of the TGF-β signaling pathway (e.g. TGF-β receptor type II and SMAD4) are indeed transported by CAFs-exosomes to cancer cells, the TGF-β signaling pathway could be reactivated, contributing to cancer progression and metastasis in this context [195].

Importantly, exosomes have great potential in clinical applications. The contents in exosomes can serve as selective cancer biomarkers in clinical biopsies, which can reflect the abnormal status of the cells and tissues [26, 28, 196]. The development of biomarkers using exosomes may lead to early cancer (relapse) detection, enable decisions on type of therapy and prevent over-treatment of patients. Also, the exosomes can be used as drug carriers with good biocompatibility, which can be easily absorbed by the target cells with little unexpected immune response [197]. The engineered exosomes with different artificial modifications are also being developed to get more efficient therapeutic effects [185]. Encouragingly, the exosome-loaded cargos acting as tumor vaccines have been used in clinical trials to activate the immune response and inhibit the cancer progression, thus showing promise to improve treatment outcome [191]. Using exosomes as cancer markers or drug carries need further optimization to obtain more stable and efficient effects. In the future, exosomes are likely to play an important role in clinical treatment.

## Data Availability

Not applicable.

## Abbreviations

TME: Tumor microenvironment
NK: Natural killer
TAMs: Tumor-associated macrophages TAMs
ECM: Extracellular matrix
ICIs: Immune checkpoint inhibitors
CAFs: Cancer associated fibroblasts
EVs: Extracellular vesicles
FAP: Fibroblast activation protein α
α-SMA: α-smooth muscle actin
FSP1: Fibroblast-specific protein 1
PDPN: Podoplanin
PDGFR: Platelet-derived growth factor receptor
myCAFs: myofibroblastic CAFs
FACS: Fluorescence activated cell sorting
ScRNA-seq: Single-cell RNA sequencing
iCAF: Inflammatory CAF
IL-6: Interleukin-6
IL-11: Interleukin-11
LIF: Leukemia inhibitory factor
CD25: CD25 antigen
FOXP3: Forkhead box P3
EMT: Epithelial-to-mesenchymal transition
EndMT: Endothelial-to-mesenchymal transition
TGF-β: Transforming growth factor-β
PDAC: Pancreatic ductal adenocarcinoma
Treg cells: Regulatory T cells
ER: Estrogen receptor
Versican: VCAN
IL-1α: interleukin-1α
IL-33: interleukin-33
SDF1: Stromal cell-derived factor 1
CXCL8: C-X-C motif chemokine ligand 8
COX-2: cyclooxygenase-2
MMPs: Matrix metalloproteinases
VEGF: Vascular endothelial growth factor
WNT2: Wnt family member 2
FGFs: Fibroblast growth factors
CXCL5: C-X-C motif chemokine ligand 5
CXCL12: C-X-C motif chemokine ligand 12
M-CSF: Macrophage colony-stimulating factor
PD-1: Programmed cell death 1
PD-2: Programmed cell death 2
PGE2: Prostaglandin E2
Cav-1: caveolin-1
CCL5: C-C motif chemokine ligand 5
CXCL10: C-X-C motif chemokine ligand 10
SATB-1: Special AT-rich sequence-binding protein-1
GJIC: Gap junctional intercellular communication
NSCLC: Non-small cell lung cancer
Cx43: connexin 43
TCA: Tricarboxylic acid
PI3K/AKT: Phosphatidylinositol 3-kinase/protein kinase B
MAPK/ERK: mitogen-activated protein kinases/extracellular signal-regulated kinase
HAPLN1: Hyaluronan and proteoglycan link protein 1
NEAA: Non-essential amino acids
MISEV2018: Minimal information for studies of extracellular vesicles 2018
ISEV: International Society for Extracellular Vesicles
sEVs: small EVs
m/lEVs: medium/large EVs
MVBs: multi-vesicular bodies
ILVs: Intraluminal vesicles
ESCRT: Endosomal sorting complex required for transport
ALIX: Apoptosis-linked gene 2-interacting protein X
VTA1: Vesicle trafficking 1
SNARE: Soluble N-ethylmaleimide-sensitive factor attachment protein receptor
MiRNAs: microRNAs
CDX2: Caudal-related homeobox 2
HOXA5: Homeobox A5
USP28: Ubiquitin-specific peptidase 28
CircRNAs: circular RNAs
CD44: CD44 molecule
CRC: Colorectal cancer
LncRNAs: long noncoding RNAs
ANXA2: Annexin A2
DNMT1: DNA methyltransferase 1
HIF1α: hypoxia inducible factor 1 subunit alpha
ADAM10: ADAM metallopeptidase domain 10
OTSCC: Oral tongue squamous cell carcinoma
MFAP5: microfibril associated protein 5
SHH: Sonic Hedgehog
ESCC: Esophageal squamous cell carcinoma
OCR: Oxygen consumption rate
SNHG3: Small nucleolar RNA host gene 3
PKM: Pyruvate kinase M1/M2
ESR1: Estrogen receptor 1
PTEN: Phosphatase and tensin homolog
GREM2: gremlin 2
PARP: Poly (ADP-Ribose) polymerase 1
CLCA4: Chloride channel accessory 4
CXCR5: C-X-C motif chemokine receptor 5
PD-L1: Programmed death-ligand 1
ALOX15: Arachidonate lipoxygenase 15
JAK1/STAT1: janus kinase 1/ signal transducer And activator of transcription 1
BCL2: BCL2 apoptosis regulator
PBX3: PBX homeobox 3
AXL: AXL receptor tyrosine kinase
SCAI: Suppressor of cancer cell invasion
CDKN1A: cyclin dependent kinase inhibitor 1A
FOSL1: FOS like antigen 1
IFN-γ: interferon-γ
LATS2: Large tumor suppressor kinase 2
YAP1: Yes-associated protein 1
EVPs: Extracellular vesicles and particles
PaCa: Pancreatic adenocarcinoma
LuCa: Lung adenocarcinoma
SIRPα: signal regulatory protein α
MART-1: melanoma antigen recognized by T cells 1
MAGEA1: MAGE family member A1
